# Prevalence of TPMT and ITPA gene polymorphisms and effect on mercaptopurine dosage in Chilean children with acute lymphoblastic leukemia

**DOI:** 10.1186/1471-2407-14-299

**Published:** 2014-04-28

**Authors:** Mauricio J Farfan, Carolina Salas, Cristina Canales, Felipe Silva, Milena Villarroel, Katherine Kopp, Juan P Torres, María E Santolaya, Jorge Morales

**Affiliations:** 1 Departamento de Pediatría, Centro de Estudios Moleculares, Facultad de Medicina, Universidad de Chile, Antonio Varas 360, Santiago, Chile; 2 Hospital Dr. Luis Calvo Mackenna, Santiago, Chile

**Keywords:** Genetic polymorphism, Acute lymphoblastic leukemia (ALL), 6-Mercaptopurine, TPMT

## Abstract

**Background:**

Mercaptopurine (6-MP) plays a pivotal role in treatment of childhood acute lymphoblastic leukemia (ALL); however, interindividual variability in toxicity of this drug due to genetic polymorphism in 6-MP metabolizing enzymes has been described. We determined the prevalence of the major genetic polymorphisms in 6-MP metabolizing enzymes in Chilean children with ALL.

**Methods:**

103 Chilean pediatric patients with a confirmed diagnosis of ALL were enrolled. DNA was isolated from whole blood and genetic polymorphism in thiopurine S-methyltransferase (TPMT) and inosine triphosphate pyrophosphatase (ITPA) coding genes were detected by polymorphism chain reaction-restriction fragment length (PCR-RFLP) assay.

**Results:**

The total frequency of variant *TPMT* alleles was 8%. TPMT*2, TPMT*3A and TPMT*3B alleles were found in 0%, 7%, and 1% of patients, respectively. For ITPA, the frequency of P32T allele was 3%. We did not observe any homozygous variant for TPMT and ITPA alleles. We also analyzed a subgroup of 40 patients who completed the maintenance phase of ALL treatment, and we found that patients carrying a *TPMT* gene variant allele required a significantly lower median cumulative dosage and median daily dosage of 6-MP than patients carrying wild type alleles.

**Conclusion:**

TMPT genotyping appears an important tool to further optimize 6-MP treatment design in Chilean patients with ALL.

## Background

Mercaptopurine (6-MP) is a highly effective chemotherapeutic agent for the treatment of childhood acute lymphoblastic leukemia (ALL), and is extensively used in therapeutic protocols worldwide [1]. Hematological and hepatic toxicities are the most common adverse effects associated with cumulative toxic plasma concentrations of 6-MP metabolites [2,3]. Pharmamacogenetics has provided a molecular approach to guide the individualization of cancer chemotherapy, reducing toxicity and increasing safety of the therapy [4]. Pharmacogenetic studies in childhood ALL have associated toxicity to 6-MP to single nucleotide polymorphism (SNP) in genes coding for 6-MP metabolizing enzymes such as thiopurine S-methyltransferase (TPMT) and inosine triphosphate pyrophosphatase (ITPA) [5,6].

TPMT is a cytosolic enzyme that catalyses the methylation of aromatic and heterocyclic sufohydroxyl groups in 6-MP and their nucleotide metabolites [7]. TPMT exhibits genetic polymorphism in all large ethnic groups, including Caucasians, Africans, African-Americans, and Asians and has been associated with high levels of 6-MP metabolites plasma level and toxicity [8]. TPMT activity exhibits monogenic co-dominant inheritance. Approximately one in 300 persons inherit two variant *TPMT* alleles and are *TPMT* deficient, and about 5%-10% are heterozygotes with intermediate enzyme activity, leading to severe and moderate to severe myelosuppression when patients are treated with conventional doses [6]. To date, more than 20 SNPs for TPMT have been described, but alleles TPMT*2 (1800462), TPMT*3A (rs1800460), and TPMT*3C (rs1142345) comprise about 80-95% of all variant alleles described so far [1-3].

ITPA is another enzyme involved in 6-MP metabolism. This enzyme catalyzes the hydrolysis of inosine triphosphate (ITP) to inosine monophosphate (IMP), protecting cells from the accumulation of harmful nucleotides such as ITP and deoxyinosine triphosphate. A C198A transversion (rs1127354) causing a Proline to Threonine replacement at codon 32 (P32T polymorphism) is the most relevant SNP determining low ITPA enzymatic activity [5,9].

The knowledge of SNP in 6-MP metabolizing enzymes and its related drug toxicity has developed more rational approaches to optimize chemotherapy in patients with ALL. Genetic variants for TPMT and ITPA differ from patient to patient and among different ethnic groups and impact individual toxicity related to 6-MP. Determination of the frequency of these genetic variants is necessary when considering the use of pharmacogenetics as a tool to improve treatment outcome. In this study, we determined the prevalence of TPMT and ITPA polymorphisms in Chilean children with ALL to help better characterize patients from Latin America because at our institution we are increasingly employing pharmacogenetic data for the design of new treatment strategies. In addition, we evaluated the association between polymorphisms and 6-MP dosage.

## Methods

### Study population

In a prospective study, between June 2009 and March 2010 we collected blood samples from 103 new patients under 18 years of age with a confirmed diagnosis of ALL, treated at the Dr. Luis Calvo Mackenna Hospital (n = 89) in Santiago, Chile, and Dr. Gustavo Fricke Hospital (n = 14) in Viña del Mar, Chile. These hospitals are members of the BFM international consortium for the treatment of childhood ALL, so patients were enrolled in ALL-IC-BFM2002, the therapeutic protocol open during the study period. As maintenance therapy, all patients received oral 6-MP 50 mg/m^2^ daily, and methotrexate (MTX) 15 mg/m^2^ weekly; both were taken in the evening on an empty stomach without milk. At the time of blood collection for the polymorphism study, all patients and/or parents provided written informed consent to participate in the study, approved by the Ethical Committee of the Dr. Luis Calvo Mackenna Hospital and Dr. Gustavo Fricke Hospital.

### DNA extraction

We collected peripheral blood samples (2–4 mL) from study participants in tubes containing sodium EDTA. A unique accession number to each sample was assigned and DNA from 200 μL of total blood was extracted by using the QIAmp DNA blood kit (Qiagen Inc., Valencia, CA). DNA was diluted in 200 μL distilled water and stored at -20°C until analysis.

### Genotyping

PCR-RFLP analyses were used to evaluate genetic polymorphism in *TPMT* (TPMT*2, TPMT*3A, TPMT*3B and TPMT*3C), *ITPA* (P32T) using methods previously described [5,10].

### TPMT activity measurement

Erythrocyte TPMT activity was measured by use of blood collected in heparinized tubes, as previously described [11]. Briefly, erythrocytes were washed with 0.9% NaCl and lysed with cold water. Erythrocyte membranes were then separated by centrifugation for 10 minutes at 12,000 × g. The lysates were stored at -80°C until analyzed. TPMT activity was normalized per milliliter of packed red blood cells (Units/mL). Erythrocyte TPMT activity was measured 30 or more days following the last erythrocyte transfusion. TPMT activity measurement was performed at The Department of Pharmaceutical Sciences, St. Jude Children’s Research Hospital (Memphis, TN) through collaboration with Dr. Mary V. Relling and co-workers.

### 6-MP dosages and laboratory data collection

The mean daily and cumulative dosages of 6-MP (in mg/m^2^/day and mg/m^2^) were calculated for the patients carrying variant and wild type alleles. Hematologic and hepatic laboratory parameters related with 6-MP toxicity were monitored every two weeks during the follow-up maintenance period and obtained from clinical charts and laboratory databases. The mean value for each test was also calculated. The documented tests were leucocytes, platelet, percentage of neutrophils, absolute neutrophils count (ANC), Aspartate Transaminase (AST), Alanine Transaminase (ALT) and total and direct bilirubin.

### Data analysis

All data were entered into an electronic database and analyzed by the χ2 test. GraphPad Prism software version 3.0 (GraphPad Software, San Diego, CA) was used for all calculations. The Wilcoxon rank sum test and 95% confidence interval for differences between median values were used to compare TPMT activities between subpopulations and the Mann–Whitney Rank Sum Test was used to compare clinical and laboratory findings with the two genotypes for continuous variables or parameters. In all tests *P* values ≤0.05 were considered statistically significant.

## Results

### Prevalence of TPMT and ITPA genetic polymorphisms in ALL patients

**Figure 1:**
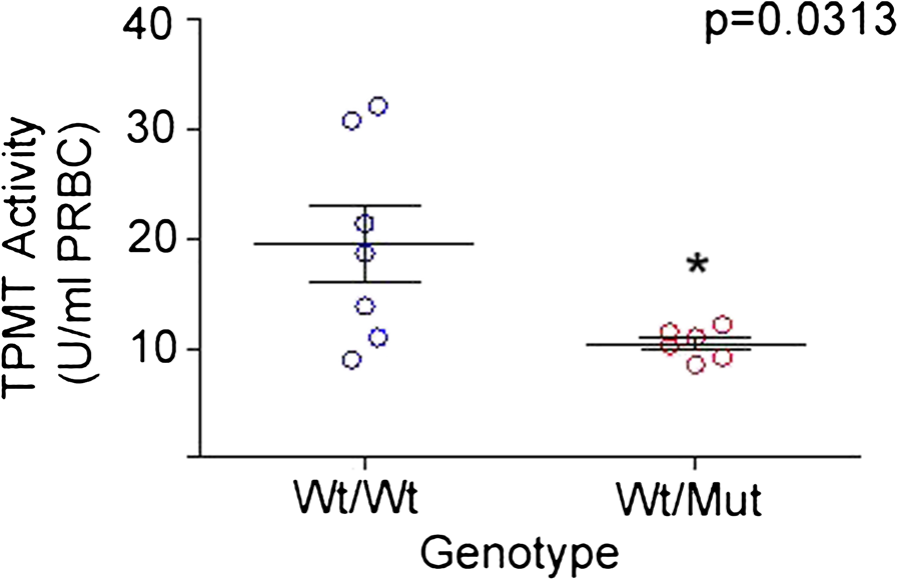
**Erythrocyte TPMT activity in Chilean patients with ALL.** TPMT enzyme activity from packed red blood cells (PRBC) in patients with a TPMT variant allele (WT/Mut; n = 6) compared to the activity identified in patients without the variant present (WT/WT; n = 7). The Wilcoxon rank sum test and 95% confidence interval for differences between median values were used to compare TPMT activities between subpopulations. *P* values ≤0.05 were considered statistically significant.

We enrolled 103 patients newly diagnosed with childhood ALL having a mean age of 8.7 years; 42/103 (41%) were male. TPMT and ITPA genetic polymorphisms were found in 8/103 (8%) and 3/103 (3%) patients, respectively. For TPMT, TPMT*3A and TPMT*3C polymorphisms were found in 7/103 (7%) and 1/103 (1%) patients, respectively. We found no homozygous variant for *TPMT* and *ITPA* genes and no TPMT*2 and TPMT*3B alleles (Table 1). To correlate these findings with the TPMT phenotype, we assayed the TPMT enzyme activity from blood erythrocytes in 6 randomly selected patients with a variant allele in the *TPMT* gene. A significantly lower TPMT activity was found in all patients with TPMT polymorphism compared to 7 patients lacking the inactivating variant (Figure 1).

**Table 1:** TPMT and ITPA genotype frequencies in 103 Chilean children with ALL

**Genotype**	**n = 103**	**%**
**TPMT**		
TPMT*1/ TPMT*1	95	92
TPMT*2/ TPMT*1	0	0
TPMT*3A/ TPMT*1	7	7
TPMT*3B/ TPMT*1	0	0
TPMT*3C/ TPMT*1	1	1
**ITPA**		
*ITPA 94 C/C*	100	97
*ITPA 94 C/A*	3	3

### Association of TPMT and ITPA genetic polymorphisms with 6-MP toxicity and dosage

**Figure 2:**
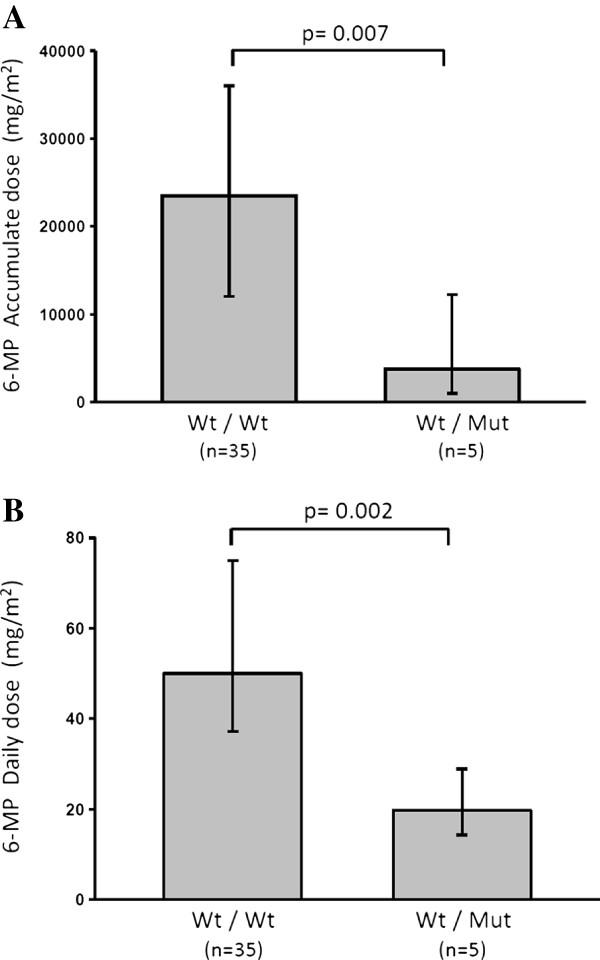
**TPMT polymorphism and 6-MP dosage in Chilean children with ALL.** Cumulative dosage **(A)** and daily dosage **(B)** of 6-MP between patients carrying a *TPMT* gene variant allele compared to patients carrying wild type alleles. The Mann–Whitney Rank Sum Test was used to compare 6-MP dosage between both groups. *P* values ≤0.05 were considered statistically significant.

To associate the presence of a TPMT and ITPA polymorphism with 6-MP toxicity and dosage, we analyzed ALL patients who completed the maintenance phase. The analysis of the subgroup of 40 ALL patients, showed that 35/40 (88%) carried wild type alleles and 5/40 (12%) carried a TPMT variant allele. No statistical differences in the measured parameters between groups were seen (Table 2). We also determined the median cumulative dosage and median daily dosage of 6-MP in the same group of patients and found that ALL patient carrying a *TPMT* gene variant allele had a significantly lower median cumulative dosage and median daily dosage of 6-MP compared to patients carrying wild type alleles (Figure 2). No differences in 6-MP toxicity or dosage were associated with ITPA polymorphisms (data not shown).

**Table 2:** Demographic profile, laboratory parameters and drug dosages during maintenance therapy of ALL children studied with and without TPMT polymorphisms

**Characteristic (median)**	**Variant alleles (N = 5)**	**Wild type (N = 35)**	***p*-Value**
Maintenance follow-up (months)	14 (11.5-20.5)	14 (12–16)	0.782
Leucocytes (×10^9^/L)	2.7 (2.35-4.23)	3.2 (2.56-4.14)	0.597
Platelet (×10^9^/L)	265.4 (±105.8)	227.7 (±70.3)	0.286
Percentage of neutrophils	56.4 (±14.5)	55.5 (±12.9)	0.887
ANC (×10^9^/L)	1.35 (1.08-2.68)	1.84 (1.30-2.20)	0.438
AST (mg/dL)	35.0 (33.7-47.5)	46.5 (30.5-54.0)	0.528
ALT (mg/dL)	28.5 (17.5-69.0)	61.0 (22.0-84.5)	0.159
Total Bilirubin (mg/dL)	0.43 (0.35-0.7)	0.59 (0.51-0.78)	0.141
Direct Bilirubin (mg/dL)	0.12 (0.11-0.22)	0.12 (0.09-0.15)	0.314

## Discussion

The latest clinical guidelines for the Childhood LLA (LLA-IC-BFM 2002) note that *TPMT* is a gene related to the antileukemic effect and side effects of 6-MP and that it is mentioned as a potential gene selected for polymorphism testing [12]. We conducted this study because of the increasing interest at our institutions in employing pharmacogenetics to refine and better individualize treatment for childhood leukemia. Although this study confirmed previous reports about the high concordance between TPMT genotype and phenotype, it is important to demonstrate these observations in our population, if we were to consider TPMT genotyping as a diagnostic tool to predict TPMT activity and its potential to develop toxicity to 6-MP at the standard dosage.

Differences in TPMT polymorphisms vary among ethnic groups, ranging from 2% to 14% prevalence. We found that allelic frequency of the most relevant TPMT polymorphisms in Chilean patients with ALL was 8% (Table 1), similar to that found in Chilean blood donors from a previous study [13], although we did not find the TPMT*2 allele. The frequency and distribution of TPMT alleles in Chile are similar to that of Hispanics in other Latin American countries. In fact, within the region, polymorphism prevalence differs only in Brazil [14,15], a finding explained by their unique racial mixture.

One of the major aspects about pharmacogenetics is the clinical consequences of one particular polymorphism in the treatment outcome. Several studies indicate that TPMT polymorphisms are associated with 6-MP toxicity or dosage [3,6,15-17]. To correlate our results with clinical findings we determined laboratory parameters and 6-MP dosage in ALL patients who completed the maintenance phase of the ALL treatment. The median daily and cumulative dosages of 6-MP in the maintenance phase were significantly lower in patients carrying variant alleles compared to wild type patients (Figure 2). These observations are in agreement with previous reports [18]. However, we did not find statistical differences in laboratory parameters related to drug toxicities, a situation that might be explained by 6-MP dosage adjustment using ANC values as clinical guidelines suggest. Overall, these observations strongly support the importance of TPMT genotyping, identifying high risk patients that can be treated with reduced dosages of 6-MP without compromising the ALL treatment.

ITPA is another enzyme involved in the 6-MP metabolism. Genetic polymorphisms in the *ITPA* gene are associated with reduced activity of the ITPA enzyme and increased toxicity to mercaptopurine. Several polymorphisms have been described for ITPA, but the P32T variant is the most common SNP [5]. The distribution of P32T polymorphism varies from 1% to 15%. In this study, we found that the prevalence of ITPA was 1% in ALL patients (Table 1), which is in agreement with the frequency reported within other Hispanic groups [19]. However, we did not find differences in 6-MP toxicity or dosage in patients carrying the P32T polymorphism compared to wild type patients, a situation might be explained by the low the prevalence for ITPA found in this study. A recent study supports the importance of this polymorphism when 6-MP dosages had been adjusted for TPMT genotype [20]. Therefore, prospective studies analyzing the involvement of TPMT and ITPA polymorphism and adverse reaction to 6-MP are warranted.

## Conclusion

We report the prevalence of the major polymorphisms in 6-MP metabolizing enzymes in Chilean patients with childhood ALL. Our data strongly support the importance of TPMT genotyping in patients with ALL to design better and more rational treatment strategies using 6-MP in children with ALL.

## Competing interests

The authors declare that they have no competing interests.

## Authors’ contributions

MJF participated in the design of the study, acquisition of data, interpretation of data and manuscript writing and final approval of the manuscript, CS carried out the genotyping, acquisition of data and interpretation of data, CC participated in the acquisition and interpretation of data, FS participated in interpretation of data and critical revision of the manuscript, MV participated in the design of the study, KK participated in the design of the study and interpretation of data, JPT participated in the design of the study and performed the statistical analysis, MES participated in the design of the study, JM participated in the design of the study, acquisition of data, interpretation of data and final approval of the manuscript. All authors read and approved the final manuscript.

## Pre-publication history

The pre-publication history for this paper can be accessed here:

http://www.biomedcentral.com/1471-2407/14/299/prepub
